# AlphaRouter: Bridging the Gap Between Reinforcement Learning and Optimization for Vehicle Routing with Monte Carlo Tree Searches

**DOI:** 10.3390/e27030251

**Published:** 2025-02-27

**Authors:** Won-Jun Kim, Junho Jeong, Taeyeong Kim, Kichun Lee

**Affiliations:** 1 Hyundai Glovis, Seoul 685-700, Republic of Korea; wjkim@glovis.net; 2 Department of Industrial Engineering, College of Engineering, Hanyang University, Seoul 133-791, Republic of Korea; jjunho1011@hanyang.ac.kr (J.J.); fasttom7@gmail.com (T.K.)

**Keywords:** deep reinforcement learning, reinforcement learning, MCTS, vehicle routing problem

## Abstract

Deep reinforcement learning (DRL) as a routing problem solver has shown promising results in recent studies. However, an inherent gap exists between computationally driven DRL and optimization-based heuristics. While a DRL algorithm for a certain problem is able to solve several similar problem instances, traditional optimization algorithms focus on optimizing solutions to one specific problem instance. In this paper, we propose an approach, AlphaRouter, which solves routing problems while bridging the gap between reinforcement learning and optimization. Fitting to routing problems, our approach first proposes attention-enabled policy and value networks consisting of a policy network that produces a probability distribution over all possible nodes and a value network that produces the expected distance from any given state. We modify a Monte Carlo tree search (MCTS) for the routing problems, selectively combining it with the routing problems. Our experiments demonstrate that the combined approach is promising and yields better solutions compared to original reinforcement learning (RL) approaches without MCTS, with good performance comparable to classical heuristics.

## 1. Introduction

In NP-hard combinatorial optimization (CO) problems, finding global optimum solutions is computationally infeasible. Instead of finding global optima, numerous heuristics have shown promising results. Despite the high effectiveness of heuristics, their application in real-life industries is often hindered by a variety of problems and uncertain information. Indeed, heuristics, which have mathematical origins, are dependent on problem formulations for proper application. However, an exact formulation of constraints is quite challenging in reality, as some constraints are rapidly changing or highly stochastic in a distributional sense. For instance, a few constraints vanish at a time, and other constraints, e.g., a newly unknown coefficient, should be estimated by an assumed distribution and a certain procedure.As real-life domains are entangled with various participants and requirements, some constraints are too complex to formulate. In such situations, particularly when simulation is possible as in a game, the approach of RL has recently attracted attention in the literature and industry.

Mostly, heuristics aim to solve one specific problem. That is to say, heuristics made for capacitated vehicle routing problems (CVRP), for example, cannot apply to bin-packing problems. To deal with versatile constraints and complex problems, the use of deep neural network architectures coupled with RL, called DRL, has recently been considered effective [1,2]. The DRL approach is flexible, as translating a problem into a reinforcement learning framework is straightforward by appropriately defining both state and reward and running computational simulations. In the long run, the ultimate goal of the DRL approach is to find a new and computational way to solve a complex problem that surpasses the performance of mathematically exact algorithms and their heuristics [3].

Nonetheless, the current stage of the performance of DRL has not achieved the performance of heuristic solvers, and there are ongoing research studies to improve its performance. Our goal is to improve DRL performance by attempting to reduce the gap between heuristic solvers and DRL. Motivated by the AlphaGo series [4,5], we propose a deep-layered network for RL equipped with the selective application of a Monte Carlo Tree Search (MCTS), a general framework applicable to various types of CO problems. We modify some components of the MCTS for application to routing domains that are different from a game.

Unlike the AlphaGo series, we observe that applying MCTS to every action choice is inefficient. To address this, we propose an entropy-based strategy for selectively applying MCTS, which is justified from an information-theory perspective and designed to enhance performance in routing problems. To the best of our knowledge, this represents a novel attempt to refine network architectures in the context of customized MCTS for routing tasks.

The introduced RL framework is quite beneficial if the resulting network is applicable to other similar problems, using the same network architecture as suggested in [1,2,6].

The contributions of our paper are threefold: (1) We propose a deep-layered neural network architecture, fitted to the routing problem, with an exact definition of states and rewards and a policy gradient using a value network. (2) We propose a new MCTS strategy, demonstrating that the integration and entropy-based selective application of our MCTS into the neural network architecture improves the solution quality. (3) We also demonstrate the usefulness of an activation function to numerically improve the solution quality. In short, the main focus of this paper is to propose an effective RL architecture with a modified MCTS strategy for routing problems and to improve search performance. Notably, although we have proposed a neural network architecture specialized to routing problems, any neural network architecture containing a policy and a value, which will be described in later sections, can be integrated into MCTS [7], and the domain, a class of routing problems in this paper, can be extended to other problems.

We organize the rest of the paper as follows: In Section 2, we provide an overview of previous works related to combinatorial optimization, routing, and MCTS. In Section 3, we briefly introduce a general formulation of capacitated vehicle routing problems and our problem’s objective. In Section 4, we expound on our proposed approach, AlphaRouter. In Section 5, we present our experimental results.

## 2. Related Works

Routing problems are among the most well-known set of problems in combinatorial optimization. A traveling salesman problem (TSP), one of the simplest routing problems, seeks to find the sequence of nodes with the shortest distance. On the other hand, a vehicle routing problem (VRP), similar to TSP, is a routing problem with the concept of depots and demand nodes. Numerous variants of VRP exist in the literature such as VRP with time windows and VRP with pickup and delivery [8]. In this paper, we focus on the capacitated VRP (CVRP), where a vehicle has a limit on its loading amount. Although some variants handle multiple vehicles, we only consider one vehicle for simplicity.

Traditionally, solutions for these problems mainly belong to two types: using math-based approaches like mixed integer programming or applying carefully designed heuristics for a specific type of problem. An example of the latter is the Lin–Kernighan heuristic [9]. In the past decade, hybrid genetic searches with advanced diversity controls have been introduced and applied to various CVRP variants successfully, greatly improving computation time and performance [10,11]. We agree that the current stage of DRL does not surpass the performance of the analytically driven heuristics but emphasize that there should be efforts to solve problems using fewer mathematics-entangled methods, such as dynamic programming and stochastic optimization.

Recent research on neural networks for routing problems can be broadly categorized into two approaches based on the type of input they use: graph modules and sequential modules. Graph modules take a graph’s adjacency matrix as an input and employ graph neural networks to solve routing problems, which are naturally suited to graph structures of routing problems [1,12]. In contrast, sequential models use a list of node coordinates as input and are designed to be compatible with certain types of exact solution inputs. In this paper, we focus on the sequential module approach.

The pointer network [6] was an early model for solving routing problems. It suggested a supervised way to train a modified Seq2Seq (sequence-to-sequence) network [13] with an early attention mechanism [14] by producing an output that is a pointer to the input token. However, a significant disadvantage of the pointer network is that one cannot obtain enough true labels to train large problems since routing problems are NP-hard. To overcome this limitation, the approach in [15] introduced the RL method for training neural networks using the famous and simple policy gradient theorem [16].

Similar to machine translation evolving from Seq2Seq to Transformers [17], routing also adopted the Transformer architecture in [18], using both the attention layer to encode the relationships between nodes and the encoding in decoder to produce a probability distribution for the most promising nodes. Replacing the internal neural network only, they kept the training of RL the same as in [15]. Due to its effectiveness in capturing complex relationships between nodes and generating high-quality solutions, attention-based DRL methods have become one of the most commonly used approaches in DRL for routing problems [18,19,20].

In addition to designing neural architectures, some works focus on the search process itself. For example, the work [2] introduced a parallel in-training search process, named POMO, based on attention network designs. The POMO algorithm assigns a different start node for several rollouts and executes multiple episodes concurrently. An episode or rollout can be understood as a process in which a vehicle travels to the next customer until all customers are visited. Among the many episodes, they selected one best solution as the final solution. Although it does not introduce any more parameters to the model, the size of the input is bound to $O\left(N^{2}\right)$ usually, where *N* is the number of total nodes in the problem. Another approach is to adjust the weights of a pre-trained model during inference to fit the model into a single problem instance as proposed in [21].

MCTS is a decision-making algorithm commonly used in games, such as chess, Go, and poker. The algorithm selects the next actions by simulating the game and updates the decision policy using data from the simulation. The original algorithm consists of four phases: selection, expansion, rollout, and backpropagation. In the selection phase, the algorithm starts at the root node and recursively goes down to a child node, maximizing the upper confidence bound (UCB) scores. This score balances exploration and exploitation in the selection process by considering the visit counts and the average value (i.e., average winning rate) gained on that node. A more detailed explanation of UCB is presented in [22].

When the selection phase ends and the current node is a leaf node, the expansion phase is executed, in which a new child node is appended to the leaf node following a specific policy named “expansion policy”. Then, the rollout phase, using “rollout policy”, simulates the game until it ends and gathers the result (i.e., win or lose). In the backpropagation phase, by backtracking the path (the sequence of selected nodes), the evaluated result from the rollout policy is updated for each node. For example, the updates increase visit counts by one and update the average win rate with the result from the rollout phase. We note that there are two different policies used in the original MCTS, but in our MCTS implementation, only the expansion policy exists, and the rollout policy is replaced by the value network. We further describe this in Section 4.

Since MCTS shares similarities with routing problems, in that sequential decision-making is involved, we adopt MCTS as an additional search strategy for routing problems. Indeed, there have been some efforts to integrate MCTS into CVRP solvers, demonstrating its potential to enhance decision-making in combinatorial optimization tasks. Upper Confidence Bounded Tree (UCT; which is an extension of MCTS)-based vehicle routing solver was suggested in [23]. An extension to HF-CVRP (Hybrid Fleet CVRP) was shown in [24]. However, these approaches applied MCTS in a non-selective manner without utilizing DRL methods.

In the game of Go, the next move is selected sequentially based on the board situation. In routing, the next node to visit is selected sequentially based on the current position and other external information, such as nodes location and demands. Thus, the similarity between Go and routing is obvious.

Neural networks have been successfully integrated with MCTS in AlphaGo [4], and AlphaGo Zero achieved even better results by introducing a self-play methodology for training the network [5]. The first AlphaGo used two different neural networks for “expansion” and “rollout”, inducing a computational burden because of many recursive calls for the “rollout” network until the end of the game. This problem is solved by AlphaGo Zero, in which one call for the value network predicts the expected result from any given state (or node). This was originally gathered in the rollout phase. In short, only one call for the value network replaced numerous calls for the rollout network, saving substantial computations. Our work adopts this idea for efficient MCTS simulation. Neural network architectures have previously been explored in reinforcement learning (RL) contexts [2,12,18].

Building on these efforts, as well as prior work applying AlphaZero [4,5], similar DRL-MCTS combinations in the pre-marshalling problem [25] or coordinated route planning [26] and some work that integrated neural network with MCTS exist not only in the area of games but also in the Q-bit routing challenge [27,28]. As far as we know, there is no research on modified MCTS specifically for routing. Thus, we aim to bridge the gap between DRL and heuristic solvers by selectively applying MCTS to VRP tasks using entropy, making it more effective for addressing various routing challenges.

## 3. Preliminaries

Before explaining our work, we introduce the formulation of CVRP with one vehicle to connect with our routing problem. We notice that TSP can be easily formulated from CVRP by modifying some conditions.

**Figure 1:**
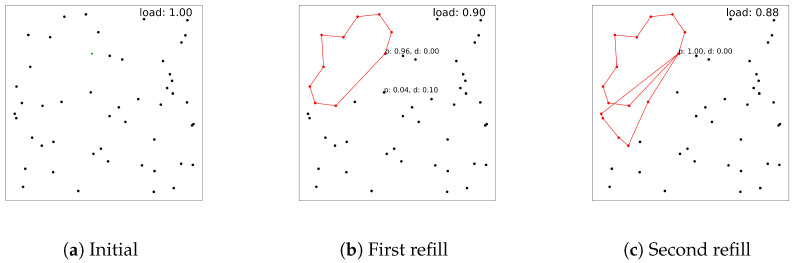
Routing results in red in dynamic load settings.

We start with a set of *n* customers, and each customer *i*, i=1,⋯,n, has a predefined positive demand quantity $q_{i}$. To fulfill the demands of the customers, the vehicle starts its route at the depot node, indexed as 0. The vehicle must visit each customer only once, and its capacity cannot be more than $Q^{max}$. In conventional settings, presumably, $Q^{max}$ is set to be sufficiently large to fulfill all customers’ demands. In reality, however, a vehicle may start with small $Q^{max}$ due to lack of information, and its load should be refilled. We observe that the problem formulation itself below cannot reflect vehicle refilling, but we aim to handle this situation as an example of the dynamic routing problem [29], using our RL approach in the next section. For example, as described in Figure 1, we present a scenario where the vehicle refills with a smaller load after its first subroute is created and solved with DRL. In the figure, dots represent customer nodes, and red dots denote the nodes visited, which are also connected by the red line. Figure 1a represents the initial setting with $Q^{max}=1$, Figure 1b shows the situation after the vehicle and is refilled with a slightly different $Q^{max}=0.9$, and Figure 1c shows the routing result with $Q^{max}=0.9$. Usually, to handle these dynamics of the environment, a complicated mathematical formulation or expert engineering techniques are needed [29]. However, with DRL, one can just adjust $Q^{max}$, which changes only one line in our implementation.

As a graph representation of the problem is common in the literature, we also represent the problem using a graph G(V,E) where V={0,1,⋯,n,n+1}, meaning all the nodes in the problem, 0 and n+1, are the same depot node. The last node n+1 is just an extra term for the ease of the formulation as the final depot of a tour. We define $\pi_{t}$ to be the node visited at time t,t≥0 with $\pi_{0}=0$ and a tour, $\pi_{t_{1},t_{2}}$, from time $t_{1}$ up to $t_{2}$ is defined as a sequence of visited nodes: for example, $\pi_{0,T}=\left[\pi_{0}=0,\pi_{1}=2,\pi_{2}=7,\ldots ,\pi_{T}=n+1\right]$, in which *T* is the last time point in the tour. The terms route and tour are used interchangeably. Additionally,

$$E=\left\{(i,j)|i,j\in V\right\}$$

refers to all the edges from all node combinations. Note that the demand of depot node $q_{0}$ is 0, meaning $q_{0}=q_{n+1}=0$. We also introduce a binary decision variable $x_{ij}$ which is 1 if there is a direct route from customer *i* to *j*, and 0 otherwise. The distance of edge (i,j) is denoted by $c_{ij}$. The cost $C(\pi_{0,t})$ is the cumulative distance calculated so far at *t*, given the sequence of visited nodes: $C\left(\pi_{0,t}\right)=c_{\pi_{0},\pi_{1}}+\cdots c_{\pi_{t-1},\pi_{t}}$, and $C(\pi_{0,0})=0$. We formulate the one-vehicle CVRP as follows:

(1) $$\begin{array}{cc} \min_{\forall x_{i,j}} & \sum_{(i,j)\in E}c_{ij}x_{ij} \end{array}$$

Subject to

(2) $$\begin{array}{ccc} \; & \sum_{\begin{array}{c} j=1,j\neq i \end{array}}^{n+1}x_{ij}=1, & \;i=1,\ldots ,n, \end{array}$$

(3) $$\begin{array}{ccc} \; & \sum_{\begin{array}{c} i=0,i\neq h \end{array}}^{n}x_{ih}-\sum_{\begin{array}{c} j=1,j\neq h \end{array}}^{n+1}x_{hj}=0, & \;h=1,\ldots ,n, \end{array}$$

(4) $$\begin{array}{ccc} \; & \sum_{j=1}^{n}x_{0j}=1, & \end{array}$$

(5) $$\begin{array}{ccc} \; & y_{i}+q_{j}x_{ij}-Q^{max}\left(1-x_{ij}\right)\leq y_{j}, & \;i,j=0,\ldots ,n+1, \end{array}$$

(6) $$\begin{array}{ccc} \; & q_{i}\leq y_{i}\leq Q^{max}, & \;i=0,\ldots ,n+1, \end{array}$$

(7) $$\begin{array}{ccc} \; & x_{ij}\in \left\{0,1\right\}, & \;i,j=0,\ldots ,n+1. \end{array}$$

We briefly present a list of equations as follows: Equation (1) is the objective of the problem, the minimization of the distance traveled by the vehicle; Equation (2) is a constraint to regularize all customers being visited only once; Equation (3) controls the correct flow of a tour, the visit sequence, by ensuring the number of times a vehicle enters a node is equal to the number of times it leaves the node; Equation (4) imposes that only one vehicle leaves the depot; and Equations (5) and (6) jointly express the condition of vehicle capacity. Note that variants for the constraints are possible, and the main reference to the above formulation is Borcinova [30]. We also notice that the finding of solution $x_{i,j}$ is equivalent to the construction of tour $\pi_{0,T}$: for example, $\pi_{1}=2,\pi_{2}=7$ represent $x_{2,7}=1$. Noticeably, in the formulation, the finding of $x_{ij}$ leads to the construction of $y_{i}$. On the route up to a visit to node j∈V, a continuous variable $y_{j}$ represents the accumulated demands and is dependent on the decision variable $x_{ij}$: for instance, on tour $\pi_{0,3}=[\pi_{0},\pi_{1},\pi_{2},\pi_{3}$], $y_{\pi_{3}}=q_{\pi_{0}}+q_{\pi_{1}}+q_{\pi_{2}}+q_{\pi_{3}}$. To migrate this formulation into TSP, one only needs to remove constraints regarding the capacity of a vehicle and the demands of customers, so that only decision variable $x_{ij}$ remains.

## 4. Proposed Network Model, AlphaRouter

In this section, we present our approach, named AlphaRouter, to solving the routing problem using both reinforcement learning and MCTS frameworks. We revise the above routing problem by adding the possibility of refilling the vehicle to reflect realistic situations. We notice that the above routing problem is unable to include the refilling action. We begin by defining the components to bring the environment into our RL problem, followed by neural network models of policy and value. We then outline our idea and implementation to adapt MCTS to the routing problem. Our overall process consists of two stages: training the neural network using reinforcement learning and combining the pre-trained network with the modified MCTS strategy to search for a better solution, meaning tour $\pi_{0,T}$ or $x_{i,j}$ in the CVRP formulation. Due to the computational demands associated with the application of MCTS, we adopt a selective application of our MCTS when ambiguity arises in choosing the next customer node that is proposed by the output distribution of the policy network, where the output distribution refers to the distribution of possible next nodes. This selective application enhances computational efficiency while maintaining the effectiveness of the MCTS strategy.

### 4.1. Reinforcement Learning Formulation

The input is denoted by $x_{i}\in R^{2}$, which represents a set of coordinates for customer *i*. The demand of a node can be included in the vector if the problem is a type of CVRP, i.e., the input for CVRP is then $\left[x_{i};q_{i}\right]\in R^{3}$, where the semicolon ; represents a concatenation operation. Also, with *n* customers, the total number of nodes is N=n for TSP, and N=n+1 for CVRP as one depot node exists. Thus, the input matrix is denoted as $Ø={\left[x_{i}\right]}_{i=1,\cdots N}\in R^{N\times 2}$ for TSP problems, and $Ø={\left[x_{i};q_{i}\right]}_{i=1,\cdots N}\in R^{N\times 3}$ for CVRP.

To bring the problem into a reinforcement learning framework, we define the state, action, and cost (inversely convertible to reward). In our work, the observation state at timepoint *t*, denoted by $s_{t}$, is a collection of the node data χ, containing coordinates and demands; the currently positioned node $\pi_{t}$; a set of available nodes to visit, denoted by $V_{t}$; and a masking vector for unavailable nodes $m_{t}\in R^{N}$, of which the $p^{th}$ element in the vector is filled with 0 if $p\in V_{t}$ and −∞ if $p\notin V_{t}$: $s_{t}=\left(\chi ,\pi_{t},V_{t},m_{t}\right)$. Though masking vector $m_{t}$ stems from the available-node set $V_{t}$ in our formulation, we intentionally add both $m_{t}$ and $V_{t}$ to the state $s_{t}$ so that the masking vector can be adjusted and redefined to reflect domain requirements just as several masking techniques are possible in Transformer [17,31,32].

We omit *t* for χ, as the node data are invariant over time in this problem: for all time points, χ stays unchanged. However, one could make node data χ varying in time depending on the domain requirement, and the proposed network model is able to handle time-varying χ. For CVRP, the current vehicle’s load, denoted by ${\mathrm{load}}_{t}=Q^{max}-y_{\pi_{t}}$, is also added to $s_{t}$: $s_{t}=\left(\chi ,\pi_{t},V_{t},m_{t},{\mathrm{load}}_{t}\right)$. The node set $V_{t}\subset V$ holds nodes, not visited yet, that are able to fulfill the demands considering ${\mathrm{load}}_{t}$.

**Figure 2:**
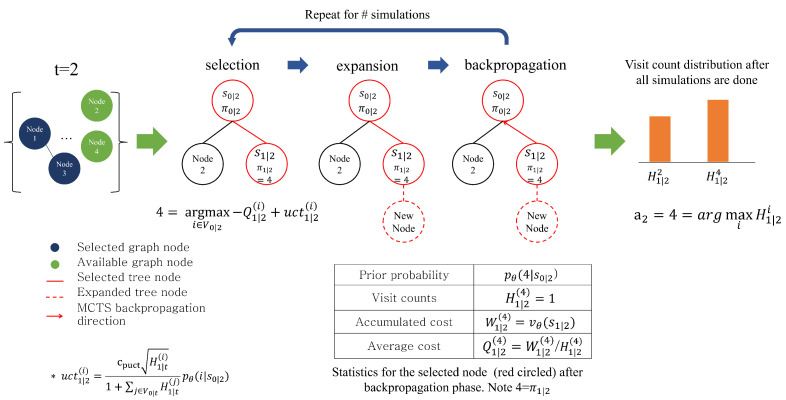
Overall process of routing using our proposed neural networks. It auto-regressively selects the next node. The encoder is executed once per episode, and the decoder is executed at every timestep *t*.

The action, denoted by $a_{t}$, is to choose the next customer node and move to it. The action in an episode, a sequence of possible states, is chosen by our policy neural network, as shown in Figure 2, which outputs a probability distribution over all the nodes given the state at *t*, $s_{t}$. We use $p_{\theta }\left(.|s_{t}\right)$ to describe the policy network output at time *t* during the episode rollout. In the training phase, the action is sampled from action distribution $p_{\theta }\left(.|s_{t}\right)$, $a_{t}∼p_{\theta }\left(.|s_{t}\right)$, as the next node to visit, meaning $\pi_{t+1}=a_{t},t\geq 0$, with $\pi_{0}=0$. The sampling operation aims to give the vehicle (or agent) a chance to explore a better solution space in our training phase. In the inference phase, however, we choose the action with the maximum probability, meaning ${\hat{a}}_{t}=\arg\max_{i\in V_{t}}p_{\theta }\left(i|s_{t}\right)$ if unvisited nodes exist, and ${\hat{a}}_{t}=n+1$ otherwise.

A value network is designed to predict the overall cost (or distance) in the episode at state $s_{t}$. This is later used in updating the MCTS tree’s statistics. We describe in detail how other components work in Section 4.2. Specifically, an episode, τ, is a rollout process in which the state and action are interleaved over t=0,1,⋯,T until the terminal state $s_{T}$ is reached: $\tau =(s_{0},a_{0},C\left(\pi_{0,0}\right),\cdots ,s_{T},a_{T},C\left(\pi_{0,T}\right))$. In this problem, the terminal state is the state in which all customers are visited and the vehicle has returned to the depot if it is CVRP. Because of the possibility of multiple refillings of the vehicle, the last time point *T* can vary in episodes of CVRP problems. For example, even when the problems have the same size (for example, n=50), the optimal solution path can vary due to different customer locations and demands. Upon reaching the terminal state, no more transitions are made, and the overall distance, $C(\pi_{0,T})$, is calculated.

### 4.2. Architecture of the Proposed Network Model

The neural network architecture of our policy network for calculating the probability distribution $p_{\theta }\left(\cdot |s_{t}\right)$ is similar to the one used in previous studies [2,18]. However, to solve the routing problem, we modify the decoder part, relying on the transformer [17]. We aim to extract meaningful, possibly highly time-dependent and complex, features that are specific to the current state while maintaining the whole node structure. We make the two networks share the same embedding vector, transformed by the current input, $s_{t}$ at time *t*. The design of the shared input transformation is a deep-layered network, consisting of an encoder and decoder, to take advantage of both the whole node structure and the current node. The structure of the two networks with the shared feature transformation is reminiscent of the architecture from the AlphaGo series [4,5] and previous related works [2,18]. In essence, the input $s_{t}$ produces the estimated probability of possible next actions via the policy network, $p_{\theta }\left(\cdot |s_{t}\right)$, and the predicted cost via the value network, $v_{\theta }\left(s_{t}\right)$. For simplicity, we denote all learnable parameters as θ, which consists of parameters from the shared transformation, those from the policy network, and those from the value network.

In detail, we explain the proposed network, dividing it into three parts: encoder in the feature transformation, decoder in the feature transformation, and policy and value. The objective of the encoder is to capture the inter-relationship between nodes. The encoder takes only the node data input χ from the $s_{t}$, passing it to a linear layer to align with a new dimensionality, $d_{e}$, via the multi-head attention layers, expressed by MHA(Q,K,V) with input tensors of query Q, key K, and value V. The output of the multi-head attention is an encoding matrix, denoted by $e\in R^{N\times d_{e}}$. Each row vector represents the $i^{th}$ node in the encoding matrix, denoted by $e_{i}\in R^{d_{e}}$. So, the currently positioned node at time *t*’s encoding is $e_{\pi_{t}}$, the embedding vector reflecting the complex and interweaved relationship with the other nodes. In summary, the encoder process is self-attention to the input node data expressed as e=MHA(Linear(χ),Linear(χ),Linear(χ)). This is repeated over several layers in the model. Relying on the idea of hidden states and current inputs in recurrent networks, we execute the encoder process once *per episode*, thereby reducing the computational burden, and use the current-node embedding and the current loading ${\mathrm{load}}_{t}$ as inputs for the decoder in a sequential manner. We provide a detailed explanation later in this section.

The decoder is responsible for revealing the diluted relationships in the encoding matrix e with additional information if it is given. Specifically, the decoder captures the relationships between the current node χ and the others. For example, let us assume that the vehicle is currently on node *i* and the current node’s embedding is $e_{i}$. Notice that we ignore time *t* in the encoding matrix since it does not change in an episode as the output of the encoder is reused over the episode once it has been executed. By using this $e_{i}$ as the query and the whole encoding matrix e as the key and value, the decoder can reveal the relationships between the current node and the others. When passing the query, key, and value, we apply linear transformations to each of them. One should note that TSP and CVRP have different inputs for the query. In CVRP, the current load, ${\mathrm{load}}_{t}\in s_{t}$, is appended to the query input, while TSP is not. While there are several layers for the encoder, we only use one layer of MHA for the decoder. A summarization of the decoder is as follows:

(8) $$\begin{array}{cc} \; & d=MHA\left(Q,K,V\right)\in R^{d_{e}},\\ & Q=\{\begin{array}{cc} \mathrm{Linear}(e_{\pi_{t}}) & \mathrm{for}\;\mathrm{TSP},\\ \mathrm{Linear}(\left[e_{\pi_{t}};load_{t}\right]) & \mathrm{for}\;\mathrm{CVRP}, \end{array}\\ & K=\mathrm{Linear}(e),\;\;V=\mathrm{Linear}(e). \end{array}$$

The policy layer and value layer are responsible for calculating the final policy $p_{\theta }\left(\cdot |s_{t}\right)$, a probability distribution on all nodes given $s_{t}$, and the predicted distance $v_{\theta }\left(s_{t}\right)$ output, respectively. We compute $p_{\theta }\left(\cdot |s_{t}\right)$ as follows with a given hyper-parameter *C* that regulates the clipping:

(9) $$\begin{array}{c} p_{\theta }\left(\cdot |s_{t}\right)=\mathrm{softmax}\left(\tanh\left(de^{T}/\sqrt{d_{e}}\right)C+m_{t}\right). \end{array}$$

To compute $p_{\theta }\left(\cdot |s_{t}\right)$, we multiply the decoder output d by the transposed encoding matrix $e^{T}$ and divide it by $\sqrt{d_{e}}$. The output goes through the tanh function, and we add the mask for the unavailable nodes using $m_{t}$. Finally, we apply a softmax operator to this result.

**Figure 3:**
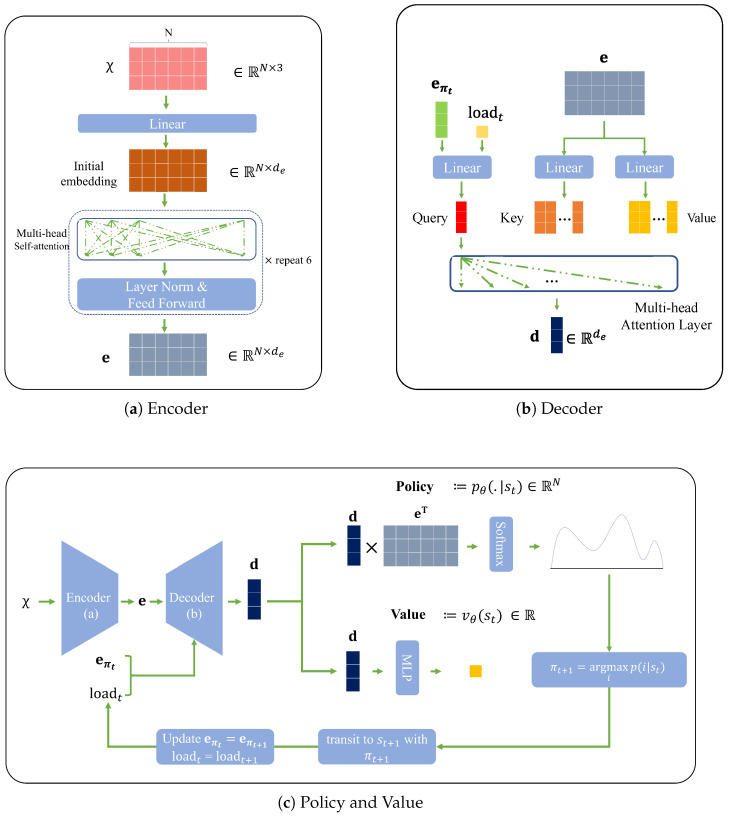
Components of the neural network.

For $v_{\theta }\left(s_{t}\right)$, we pass the same decoder output d to two linear layers of which the shape is similar to the usual feed-forward block in the transformer: $v_{\theta }\left(s_{t}\right)=\mathrm{Linear}\left(\sigma \left(\mathrm{Linear}\left(d\right)\right)\right)$, in which σ(·) is an activation function such as ReLU and SwiGLU [33,34]. A diagram for each neural network design is presented in Figure 3.

When training the model for an episode, the encoding process is only required once as the input of the encoder (the coordinates of nodes) is fixed along the rollout steps. The decoder, on the other hand, takes the inputs that change over time, i.e., the current node and current load. Thus, on first execution of the model, we execute both the encoder and the decoder. After the first execution, we execute only the decoder and policy and value parts, saving considerable computations. The encoder and decoder share the same parameters, while the policy and value networks do not. Figure 2 explains the overall process.

Additionally, we intentionally exclude residuals in the encoder layers, as we have observed that, unlike the original transformer and its variants, residual connections greatly harm the performance of the model. Another variation we have added to the previous model is the activation functions. Recent studies on large language models (LLMs) exploited different activation functions for their work. We take this into account and test SwiGLU activation, just as Google’s PaLM did in [35]. We report the results in Section 5.

### 4.3. Training the Neural Network

To train the policy network θ, we use the well-known policy gradient algorithm, ‘reinforce with the baseline’ [36]. This algorithm deals with high-variance problems prevalent in policy gradient methods by subtracting a specially calculated value, called the baseline. This algorithm collects data during an episode and updates the parameters after each episode ends. For $C(\pi_{0,T})$, the distance traveled by the vehicle following the sequence $\pi_{0,T}$, the policy network aims to learn a stochastic policy that outputs a visit sequence with a small distance over all problem instances. The gradient of the objective function for the policy network is formulated as follows:

(10) $$\begin{array}{cc} \; & \nabla J_{\theta }\left(\pi \right)\propto E_{\pi ∼p_{\theta }\left(\cdot |s\right)}\left[\left(C\left(\pi_{0,T}\right)-b\left(s\right)\right)\nabla \log p_{\theta }\left(\pi |s\right)\right], \end{array}$$

$$\begin{array}{cc} \; & \mathrm{where}\;p_{\theta }\left(\pi |s\right)=p_{\theta }\left(\pi_{0}|s_{0}\right)\prod_{k=1}^{T-1}p_{\theta }\left(\pi_{k}|s_{k},\pi_{k-1}\right) \end{array}$$

in which b(s) is a deterministic greedy rollout from the best policy trained so far as a baseline in order to reduce the variance of the original formulation [18]. After training model parameter θ for an epoch, we evaluate it with a validation problem set, setting b(s) as the evaluated cost in the validation. One can think of this procedure as the training-validation mechanism in general machine learning.

The mere use of a baseline incurs additional computational costs arising from the rollouts of several episodes, being an expensive procedure. To alleviate this burden, we introduce a value network, $b\left(s\right)=v_{\theta }\left(s\right)$, instead of the greedy rollout baseline.

The value network’s objective is to learn the expected cost at the end of the episode from any state during episode rollout. We keep track of the value network’s output throughout a rollout and train the network with the loss function

(11) $$\begin{array}{c} L_{v}\propto \sum_{t=0}^{t=T}{\left(C\left(\pi_{0,T}\right)-v_{\theta }\left(s_{t}\right)\right)}^{2} \end{array}$$

As in the POMO approach [2], we test the baseline using the average cost over a batch of episodes in addition to the baseline using value network $v_{\theta }\left(s_{t}\right)$. For instance, we calculate the baseline as the mean of all 64 episodes as a batch size, representing the number of concurrent episode runs. This value network is also used in the MCTS process described in the next section. Since our model shares the parameters in the encoder and decoder between the policy network and the value network, an update in the value network affects the parameters in the policy network with the gradient of the final loss as follows:

(12) $$\begin{array}{c} \nabla L\propto \nabla J_{\theta }\left(\pi \right)+\nabla L_{v}. \end{array}$$

### 4.4. Proposed MCTS for the Routing

The main idea of MCTS is to improve the solutions, good in general, of trained policy and value networks to be problem specific by further investigating possible actions. In essence, without MCTS, we make a transition from $s_{t}$ to $s_{t+1}$ by taking action $a_{t}$, which is the output from the policy network only. However, in our proposed MCTS as described in Figure 2, we select the next node by considering costs, which is the output of the value network, in addition to the prior probabilities from the policy network. In addition, we selectively apply the MCTS at time *t* when the highest probability from the current policy network fails to dominate, meaning actions other than the highest-probability action need to be considered. In practice, when the difference between the highest probability and the 5th highest probability is less than 0.75, we apply the MCTS, expounded below.

**Figure 4:**
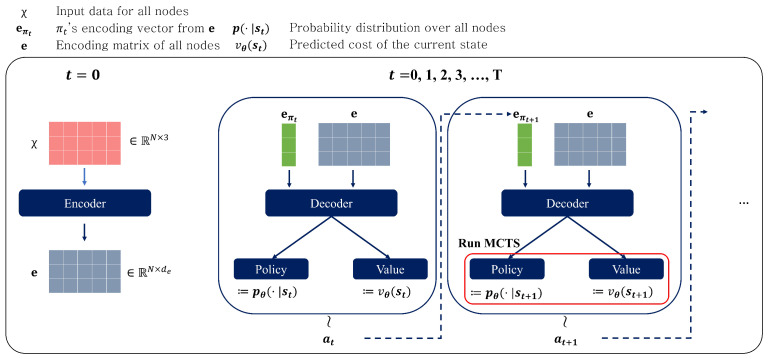
An overall process of transition using MCTS depicts a situation in which MCTS is run at t=2, and some simulation iterations are performed.

MCTS comprises three distinct phases: selection, expansion, and backpropagation. They iterate with a tree, initialized by the current node $\pi_{t}$ and updated as iterations continue, for a given number of simulations, denoted by ns as the total number of the MCTS iterations. At each iteration, the tree keeps expanding, and the statistics of some nodes in the tree are updated. As a result, a different set of tree node paths is explored throughout the MCTS iterations. Figure 4 describes an MCTS procedure in which a few MCTS iterations are run. Given time *t*, we use $s_{k|t}=\left(\chi ,\pi_{k|t},V_{k|t},m_{k|t}\right)$ to represent a tree node positioned at level *k*. The definition of $s_{k|t}$ is the same as $s_{t}$ with the only difference being that $s_{k|t}$ represents inner time step *k* temporarily used in MCTS selection. Thus, in an MCTS iteration, with fixed *t*, level *k* advances as different levels are selected in the selection phase.

In the beginning, we initialize the root tree node $s_{0|t}$ with $s_{t}$, meaning that MCTS starts from $s_{t}$, therefore the vehicle position in $s_{0|t}$ is the same as the position at *t*, $\pi_{0|t}=\pi_{t}$. To describe the MCTS phases, we introduce new notations: for the $i^{th}$ customer (or depot) node, $H_{k|t}^{\left(i\right)}$ denotes an accumulated visit count, and $W_{k|t}^{\left(i\right)}$ an accumulated total cost, both at the $k^{th}$ level of the tree. Then, we compute the ratio $Q_{k|t}^{\left(i\right)}=W_{k|t}^{\left(i\right)}/H_{k|t}^{\left(i\right)}$, called the Q-value. The Q-value, $Q_{k|t}^{\left(i\right)}$, for the *i* node represents an averaged cost at the level *k*. We normalize all Q-values in the simulation by min-max normalization.

In the selection phase, given the current MCTS tree, we recursively choose child nodes until we reach a leaf node in the tree. For instance, at the $k^{th}$ level of the tree node, among possible nodes, denoted by $V_{k|t}$, we select the next node at $s_{k|t}$ according to Equation (13), thereby moving to a tree node at the $k+1^{th}$ level:

(13) $$\begin{array}{cc} \pi_{k+1|t}={\hat{a}}_{k|t}= & \arg\max_{i\in V_{k|t}}-Q_{k+1|t}^{\left(i\right)}+\frac{c_{\mathrm{puct}}\sqrt{H_{k+1|t}^{\left(i\right)}}}{1+\sum_{j\in V_{k|t}}H_{k+1|t}^{\left(j\right)}}p_{\theta }\left(i|s_{k|t}\right), \end{array}$$

in which hyper-parameter $c_{\mathrm{puct}}$ adjusts the contribution of the policy-network evaluation $p_{\theta }\left(\cdot |s_{k|t}\right)$ in comparison with the negative of averaged cost $Q_{k|t}^{\left(i\right)}$ for node *i*. Let us use *ℓ* to denote the leaf level in the tree in the selection phase. We obtain an inner *state* path $s_{0,\ell |t}=\left[s_{0|t},\cdots ,s_{\ell |t}\right]$ and an inner *node* path $\pi_{0,\ell |t}=\left[\pi_{0|t},\pi_{1|t},\cdots ,\pi_{\ell |t}\right]$. Then, the total *node* path from time 0 to the level *ℓ* becomes a concatenation of outer-path nodes $\pi_{t-1}$ and inner-path nodes $\pi_{0:\ell |t}$: $\left[\pi_{t-1};\pi_{0:\ell |t}\right]$. The selection phase continues until no more child nodes are available to traverse from the current position, meaning that the node is a leaf node in the tree. In Figure 2, for instance, node 4 is selected, highlighted in red, from the root node in the first selection phase, and ℓ=1. Note that, in the next MCTS iteration, the selection phase starts again from the root node $s_{0|t}$ again, not from the leaf node selected from the previous iteration.

After the selection phase, the expansion phase starts, updating the MCTS tree by expanding new child nodes in $V_{\ell |t}$ at node $\pi_{\ell |t}$ and moving to the backpropagation phase. Note that in the early stages of the MCTS iterations, the tree may not have expanded enough to select a terminal node, meaning $V_{\ell |t}\neq \emptyset ,\ell <T-t$. As the MCTS iteration advances, the tree expands enough so that the final selected node from the selection phase, $\phi_{\ell |t}$, becomes the terminal node, $V_{\ell |t}=\emptyset ,\ell =T-t$, meaning that routing has ended with no available node to move to. In the latter case, the MCTS iteration continues until it reaches ns in order to explore a variety of possible node paths.

Finally, in the backpropagation phase, tracing back $\pi_{0:\ell |t}$, we update $H_{k|t}^{\left(i\right)}$ and $W_{k|t}^{\left(i\right)}$ for all selected tree nodes in k∈[ℓ,ℓ−1,⋯,0] and all selected customer nodes $i\in \pi_{0:\ell |t}$. Specifically, the update follows the rule below:

(14) $$\begin{array}{c} H_{k|t}^{\left(i\right)}=H_{k|t}^{\left(i\right)}+1, \end{array}$$

(15) $$\begin{array}{c} W_{k|t}^{\left(i\right)}=\{\begin{array}{cc} W_{k|t}^{\left(i\right)}+C\left(\left[\pi_{0,t-1};\pi_{0:\ell |t}\right]\right), & \;\ell =T-t,\\ W_{k|t}^{\left(i\right)}+v_{\theta }\left(\phi_{\ell |t}\right), & \;\ell <T-t. \end{array} \end{array}$$

As the MCTS iteration continues, the selected leaf node can be either a terminal node (ℓ=T−t), meaning that the routing has ended, or a non-terminal node (ℓ<T−t). In the former case, $W_{k|t}^{\left(i\right)}$ determines the cost by evaluating the selected path of customer nodes, $C(\left[\pi_{0,t-1};\pi_{0:\ell |t}\right])$. However, in the latter, we use the predicted distance $v_{\theta }\left(\phi_{\ell |t}\right)$. This is possible, as we train the value network $v_{\theta }\left(\cdot \right)$ to predict the final distance at any state following Equation (11). In updating accumulated total cost $W_{k|t}^{\left(i\right)}$ as in Equation (15), we obtain the predicted cost using $v_{\theta }\left(\phi_{\ell |t}\right)$ at the final selected node $\phi_{\ell |t}$, then by greedily selecting the next customer node until routing finishes.

When finishing all simulations, we collect a visit-count distribution from the $s_{0|t}$’s child nodes and choose the most visited node as $a_{t}$ for the next node to visit in the rollout:

(16) $$\begin{array}{cc} \; & \pi_{t+1}={\hat{a}}_{t}=\arg\max_{i\in V_{0|t}}H_{1|t}^{\left(i\right)}. \end{array}$$

Algorithm 1 summarizes the overall process of our MCTS. Additionally, application of the MCTS is computationally expensive, making it impractical for real-world use. For each moment in time *t*, the entropy of the probability distribution $p_{\theta }\left(\cdot |s_{t}\right)$ is computed by the formula $-\sum_{j=0}^{l}p\left(\pi_{j|t}|s_{t}\right)\log p\left(\pi_{j|t}|s_{t}\right)$. We find that most $p_{\theta }\left(\cdot |s_{t}\right)$ outputs have low entropy, meaning the highest probability, $\max_{i}p_{\theta }\left(\cdot |s_{t}\right)$, dominates other values. Our idea is that we selectively apply our MCTS to the rollout when $\max_{i}p_{\theta }\left(\cdot |s_{t}\right)$ fails to dominate, i.e., when the difference between the highest probability and the fifth highest probability is less than 0.75. We empirically obtain a strategy to improve solution quality via computation time trade-off.
**Algorithm 1** Overall simulation flow in MCTS**Require:** $s_{0|t}$: root state initialized by $s_{t}$, $p_{\theta }$: trained policy network, $v_{\theta }$: trained value network, ns: number of simulations to run
_1:_ Initialize the MCTS tree by $s_{0|t}$_2:_ **while** i<ns **do**_3:_     $\phi_{\ell |t}$, $s_{0:\ell |t}$, $\pi_{0:\ell |t}$ = Select($s_{0|t}$)          ▹ A leaf node in the MCTS tree is chosen_4:_     Expand($p_{\theta }$, $\phi_{\ell |t}$) ▹ Expand the MCTS tree from the leaf node using available nodes $V_{\ell |t}$_5:_     **if** $V_{\ell |t}=\emptyset$ **then**                            ▹ The selection reached the terminal node_6:_                    $c=C(\left[\pi_{0,t-1};\pi_{0:\ell |t}\right])$                                       ▹ (Equation (15))_7:_     **else**_8:_         $c=v_{\theta }\left(\phi_{\ell |t}\right)$       ▹ Use the predicted cost for non-terminal leaf nodes_9:_     **end if**_10:_     Backpropagate($s_{0:\ell |t}$, *c*)_11:_     i=i+1_12:_ **end while**_13:_ **return**$\arg\max_{i\in V_{0|t}}H_{1|t}^{\left(i\right)}$

We present the pseudo-code for each MCTS phase in Algorithm 2. We highlight the modifications made to adapt MCTS to the routing problems. Firstly, we apply min-max normalization to the Q-value calculated during the entire search phase. Since the Q-value range is in [0,∞), which is equal to the range of cost (distance), this can cause a computational issue as the term $\frac{c_{puct}\sqrt{H_{k+1|t}^{\left(i\right)}}}{1+H_{k+1|t}}p_{\theta (i|s_{k|t})}$ typically falls within the range [0,1]. Using a naïve Q-value could lead to a heavy reliance on the Q-value when selecting the child node because of the scale difference. To apply min-max normalization to the Q-value in the implementation, we record the maximum and minimum values in the backpropagation phase. Secondly, to minimize the distance, we negate the Q-value so that the search strategy aims to minimize distance. In the pseudo-code, the STEP procedure, which we do not include in the paper due to its complexity, accepts the chosen action as input and processes the state to transit to the next state. Internally, we update the current position of the vehicle as the chosen action in addition to the current load of the vehicle if the problem is CVRP. In addition, the mask for unavailable nodes, $m_{t}$, is updated to prevent the vehicle from returning to visited nodes.
**Algorithm 2** List of functions in MCTS.**Require:** $c_{puct}$ = 1.1: hyper-parmeter
_1:_ **function** select($s_{0|t}$)_2:_     $node\leftarrow s_{0|t}$_3:_     $s_{0:\ell |t}$ = [$s_{0|t}$]_4:_     $\pi_{0:\ell |t}$ = [$\pi_{0|t}$]_5:_     k=0_6:_     **while** node has child **do**_7:_         $\pi_{k+1|t}=\arg\max_{i\in V_{k|t}}-Q_{k+1|t}^{\left(i\right)}+\frac{c_{\mathrm{puct}}\sqrt{H_{k+1|t}^{\left(i\right)}}}{1+\sum_{j\in V_{k|t}}H_{k+1|t}^{\left(j\right)}}p_{\theta }\left(i|s_{k|t}\right)$▹ (Equation (13))_8:_         Append $\pi_{k+1|t}$ to $\pi_{0:\ell |t}$_9:_         $node=s_{k+1|t}$ is updated with the child node selected_10:_         Append node to $s_{0:\ell |t}$_11:_         k=k+1_12:_     **end while**_13:_     $\phi_{\ell |t}=node$                                                                             ▹ Also, ℓ=k_14:_     **return** $\phi_{\ell |t}$, $s_{0:\ell |t}$, $\pi_{0:\ell |t}$_15:_ **end function**_16:_ **function** expand($p_{\theta }$, $\phi_{\ell |t}$)_17:_     **for all** $i\in V_{\ell |t}$ **do**_18:_         *s*, cost, done = step(*i*, $\phi_{\ell |t}$) ▹ Run STEP with the leaf node’s state for the given *i*_19:_         create a new child node and assign *s* as the state_20:_         append the child node to $\phi_{\ell |t}$_21:_     **end for**_22:_ **end function**_23:_ **function** backpropagate($s_{0:\ell |t}$, $\pi_{0:\ell |t}$, *c*)_24:_     get [ℓ,ℓ−1,⋯,0] from $s_{0:\ell |t}$_25:_     **for all** *k*∈ [ℓ,ℓ−1,⋯,0] and $i\in \pi_{0:\ell |t}$ **do**▹*k* denotes a level from the leaf to the root_26:_         $H_{k|t}^{\left(i\right)}+=1$_27:_         $W_{k|t}^{\left(i\right)}+=c$_28:_         $Q_{k|t}^{\left(i\right)}=\frac{W_{k|t}^{\left(i\right)}}{H_{k|t}^{\left(i\right)}}$_29:_         Normalize $Q_{k|t}^{\left(i\right)}$_30:_     **end for**31:**end function**


## 5. Experiments

At first, we generate problems by constructing *N*, the number of all nodes, random coordinates of which each coodinate is uniformly distributed in range [0,1]. For CVRP, we set the first node as the depot node. In addition, the demand of each customer, $q_{i}$, is assigned an integer between 1 and 10, scaled by 30, 40, and 50 for the problem size (*n*) 20, 50, and 100, respectively. We also apply POMO [2] in our training, setting the pomo size as the number of customer nodes. However, in the inference phase, we exclude POMO, as the utilization of MCTS is infeasible. Our implementation, available at the github https://github.com/glistering96/AlphaRouter (accessed on 19 February 2025), is built on Pytorch Lightning [37] and Pytorch [38]. For the setting of MCTS, we set $c_{\mathrm{puct}}$ as 1.1, and vary the total number of simulations, ns, by 100, 500, and 1000. We measure the performance on 100 randomly generated problems as described above. In all tables presented in this section, “dist” refers to the average total distance traveled across all instances, while “time” represents the average inference time. For the selective MCTS approach, the “time” includes both the DRL inference time and the additional computational cost incurred by the selective application of MCTS.

For the encoder and decoder settings, the size of each head’s dimension is 32 with 4 heads, summed up to the embedding dimension $d_{e}=128$, and 6 encoder layers are used. We train the model for 300 epochs with batch size 64, and 100,000 episodes. Note that this batch size is the number of parallel rollouts in training, meaning that 64 episodes are simultaneously executed. We fix the clipping parameter *C* to 10. We use Adam [39] with a learning rate of $1\times 10^{-4}$, *eps* of $1\times 10^{-7}$, and *betas* ∈{0.9,0.95} without any warm-up or schedulings. For fast training, we use 16-bit mixed precision.

We conduct the experiment on a machine equipped with i5-13600KF CPU, RTX 4080 16 GB GPU, and 32 GB RAM on Windows 11. For heuristic solvers in the experiment, we use the same machine except with a WSL setting on Ubuntu 22.04.

### 5.1. Performance Comparison

In this section, we compare the performance of the proposed MCTS-equipped model with that of some heuristics for the two routing problems, TSP and CVRP. The baseline models for TSP are heuristic solvers, LKH3 [40], Concorde [41], Google’s OR Tools [42], and Neareset Insertion strategy [43]. These heuristic solvers, developed by optimization experts, serve as benchmarks for assessing optimization capabilities in solving routing problems. For Google’s OR Tools, we add a guided-search option, and the result reported here is better than the result reported in the previous research [2,18]. For a fair comparison, the time limit for OR Tools is set to be similar to the longest MCTS runtime, ns=1000, for each *n*.

For comparison, additionally, we denote the proposed model without the MCTS strategy as an attention model (AM) that leverages solely the proposed neural network without MCTS. When integrating the MCTS strategy with the network model, we vary the number of simulations to investigate its impact on performance. We evaluate one case using 100 simulations randomly generated by the same generation strategy employed during training. The comparative results of TSP and CVRP problems are summarized in Table 1 and Table 2, respectively. The column named ns represents the number of simulations in the MCTS strategy, and ns=0 denotes the AM result without the MCTS strategy. Thus, the ‘DRL’ method includes the proposed method and the AM method. The column named ‘baseline’ represents the different baseline b(s) used in (10), in which “mean” represents the mean-over-batches baseline, and ‘value’ represents the baseline with the value network, $v_{\theta }$. The baseline term here differs from the heuristic baselines reported in the tables. The best results in the experiment cases of the DRL methods are presented in bold.

**Figure 5:**
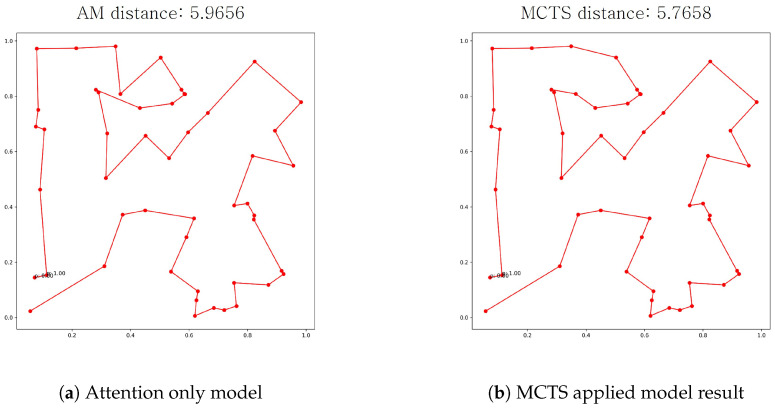
Visualization of the two methods’ routing results in red on the same test data.

For TSP, none of the records from AM, denoted by ns=0, are presented in bold, meaning that the MCTS application improves solution quality. For CVRP, some records using AM are bold, but the best records are all from the cases with MCTS. We provide the visualization of the two different methods results in Figure 5 for a clearer understanding of the effectiveness of MCTS. Visually and quantitatively, the solution in Figure 5b of the proposed model is better than that in Figure 5a of the AM. As for the scalability analysis, our results indicate that as the problem size increases, the runtime exhibits an upward trend. As shown in our table, the computational cost increases substantially when scaling from smaller instances (e.g., 20 nodes) to larger instances (e.g., 100 nodes). This trend is expected, as larger problem sizes inherently introduce greater complexity in both the DRL-based inference process and the selective MCTS execution. Furthermore, our results demonstrate that as the number of simulations (ns) increases, the runtime also increases correspondingly. This observation aligns with the expected behavior, as a higher number of MCTS iterations leads to a more extensive search process, thereby incurring an additional computational overhead.

The results reveal that while the application of the MCTS contributes to performance enhancement compared to the ones without the MCTS, it still falls short of the performance achieved by the heuristic models, as other research shows [2,18]. Contrary to our expectations, an increase in the number of simulations does not consistently lead to solution improvement, i.e., a decrease in distance. The analysis indicates the lack of a discernible relationship between the number of simulations and the resulting distance. Specifically, for problems with a size of 50, Pearson’s correlation coefficient between the two is −0.72, and for the case of CVRP with a size of 100, it is −0.47. In other cases, correlation scores are generally low, below 0.2.

**Figure 6:**
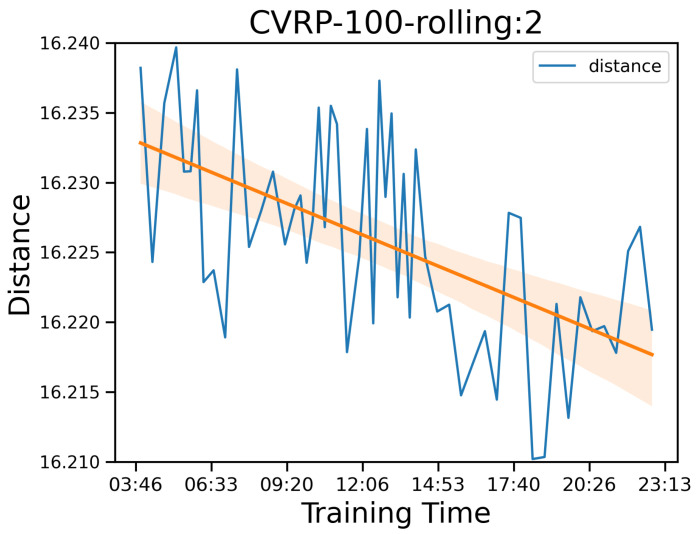
Training score with the distances in blue and the fitting in orange according to training time after 300 epochs on the CVRP-100 problem smoothed on a window size of 2.

In addition, the MCTS strategy induces little runtime overhead compared to the method without MCTS, AM. For CVRP problems, the average runtimes of the proposed method are considerably shorter than those of the LKH3 method. However, the runtime increases according to the problem size *n*. Our explanation is that, as the problem gets bigger, some large problems are hard to solve only by the probability outputs from $p_{\theta }$, therefore utilizing MCTS more. We argue that to improve the solution quality of AM, numerous samples of solutions, which take a huge amount of time to generate, are required. This result is also shown in the experiment results from [2]. Also, we point out that training the network with more learning epochs to lower a small amount of distance takes quite a long time. For example, to lower about 0.015 in distance by training the network after 300 epochs, we need approximately 24 h as described in Figure 6, in which the orange line is the regressed line over the observations. However, with MCTS, we can consistently obtain better results within a few seconds, and the method is deterministic, unlike sampling methods. Nonetheless, we believe there still is room to improve the runtime of MCTS. The heuristic solvers are written in C, while our MCTS is written in Python 3.10.12, which is very slow. Implementing MCTS in C++ with parallelism should decrease the runtime.

To statistically confirm the effectiveness of the proposed MCTS, we include paired *t*-test results for two different cases. Table 3 reports the test results with the same conditions, including activation function and baselines. The records in the AM column follow the format “activation-baseline” and the records in the MCTS column follow “activation-baseline-MCTS simulation number”. In this setting, the test shows that applying the MCTS improves the solution except for TSP-20 and CVRP-50. We suspect that for relatively small-sized problems, relying on policy network only (AM) can be good enough, but if the problem gets bigger, introducing the MCTS can result in better solutions. Table 4 shows the test results regardless of the conditions, and thus the lowest *p*-value for each problem type and size. Application of the MCTS appears worthwhile for a given problem type, even when change in the activation and baseline is allowed.

**Figure 7:**
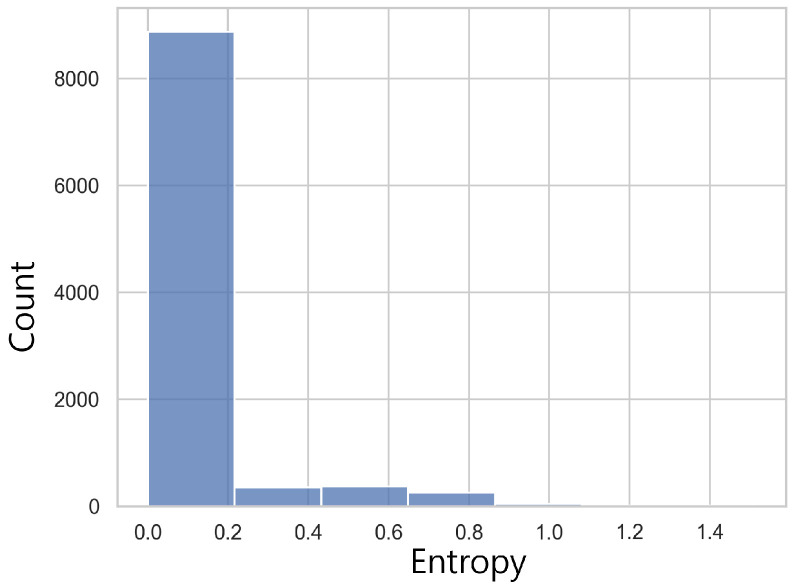
Histogram of entropies of policy network $p_{\theta }\left(\cdot \right)$ for TSP with size 100.

We also show the entropy of $p_{\theta }\left(\cdot \right)$ for all test cases in TSP with size 100 in Figure 7. We find that 86% of entropies are below 0.1, meaning that the outcomes of $p_{\theta }\left(\cdot \right)$ are dominated by a few suggestions and mere application of $p_{\theta }\left(\cdot \right)$ might lead to local optimal. Therefore, while controlling time overhead, applying MCTS selectively is a good strategy. We show the evaluation result for selective MCTS application in the ablation analysis in Section 5.2.

### 5.2. Ablation Study

In this section, we present some ablation results for the activation function and baseline. We aggregate the results based on the activation function used and then calculate the mean and standard deviation of the score over all settings, i.e., AM, MCTS-100, MCTS-500, and MCTS-1000, and the results are described in Table 5.

We can easily see that as the problem size increases, SwiGLU produces shorter distances overall than ReLU. Also, we notice that as the problem size gets bigger, the difference between ReLU and SwiGLU becomes more apparent. For example, for CVRP problem types, when the problem size is n = 20, the difference in the distances is about 0.01, while at *n* = 100, the value reaches around 0.07. The scalability of SwiGLU is much better than ReLU.

For the baseline, we suggest two different approaches: mean over batches and a value-net-based approach. This baseline is used in (10) as b(s). We calculate the mean and standard deviation of the distances over all settings based on the type of baseline used, like we did in the activation function analysis, and the result is described in Table 6. Surprisingly, the mean baseline approach dominates over the value net baseline except for CVRP with a problem size 100, which is the hardest problem in the settings. We presume that if the problem becomes complex, the value net baseline may perform better than the mean baseline. For instance, CVRP with a time window and pick-up may be solved better with the value net baseline.

We also report the non-selective MCTS experiment results here in Table 7 and Table 8. The readers should be aware that the hardware environment here is a little different from the results in the experiment section. For non-selective MCTS, we run the experiment on a workstation shared among others that runs on Linux with RTX 4090 24GB and Intel Xeon w7-2475X. Therefore, the runtime recorded in the tables below cannot be directly compared to the results in the table from the experiment, but they do indicate the trends in runtime. Despite different hardware settings and computing settings, the difference in runtime between selective MCTS and non-selective MCTS is substantial, and suggests that selective MCTS is meaningful.

We can see that compared to the selective MCTS results from Table 1, a huge amount of runtime is required for MCTS. Also, the bold values for each group are almost the same or a little bit better with the selective MCTS strategy.

We can also observe similar trends on CVRP. One can easily see the huge deviation in runtime for ns=1000 records in both TSP and CVRP. Application of MCTS to every transition does not fully account for this deviation, and the shared resource characteristic of the workstation may have also affected it. Therefore, considering the runtime and performance, selective MCTS is the better approach.

### 5.3. Mcts Application Rule

In this section, we report two different methods for applying MCTS. The first is the introduced method, which uses the difference between the highest probability and the fifth-highest probability (diff_cut), and the second is to use the entropy of the policy network directly (ent_cut). For each method, we compare how different pivot numbers affect the performance and runtime. By adopting the results from activation, we only use the SwiGLU activation for this test, and for better visibility of the changes, we focus on the problem with size 100. The difference method’s results are reported in Table 9 and Table 10, and the entropy method’s results are reported in Table 11 and Table 12.

**Figure 8:**
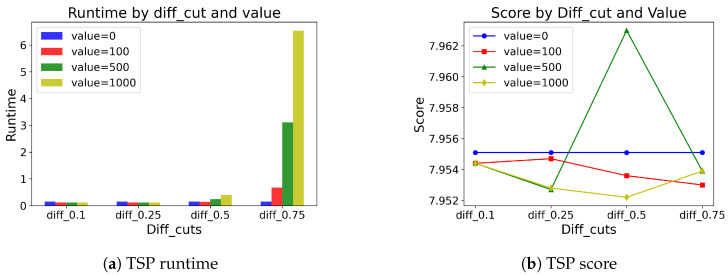
Visualization of TSP results on runtime and score.

**Figure 9:**
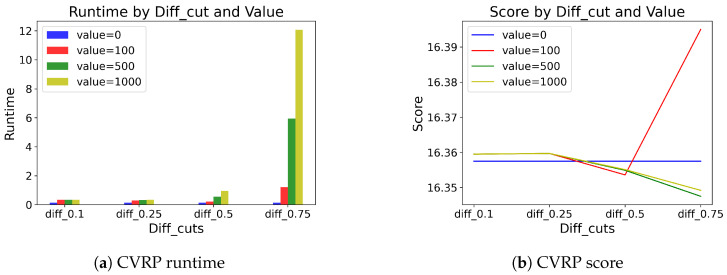
Visualization of CVRP results on runtime and score.

Table 9 and Table 10 show the results of different pivot values selected in the MCTS application. For example, diff_cut = 0.25 denotes that we apply MCTS when the difference between the highest probability and the fifth-highest probability is less than 0.25. If the difference is large, suggestions from $p_{\theta }$ are convincing, and if the difference is small, relying on $p_{\theta }$ only is not enough. Therefore, the higher the diff_cut is, the more MCTS is applied, and vice versa. For both problem types, not much difference is observed when the diff_cut < 0.5. However, if the diff_cut ≥ 0.5, we observe that applying MCTS does make a greater difference. We also find that SwiGLU coupled with the value baseline outputs generally produces better solutions than with the mean baseline outputs. Moreover, for the sake of intuitive visualization, we illustrate the results of SwiGLU coupled with the value baseline outputs for both TSP and CVRP problems in Figure 8 and Figure 9.

Table 11 and Table 12 show the results of the different pivot values selected for MCTS application using entropy value of $p_{\theta }$. For example, we only apply MCTS when the entropy of $p_{\theta }$ exceeds the given pivot value. Thus, the higher the ent_cut is set, the less MCTS is applied. Overall, the performance of ent_cut is not better than the diff_cut method in general. In addition, we can observe that the value baseline generally works better with MCTS than the mean baseline in CVRP. We suspect that the diff_cut method leads to a narrower search boundary than the ent_cut method, which is similar to the trust region in gradient descents.

In summary, the heavy application of MCTS may result in unsatisfactory performance, increasing the runtime significantly. Finding the optimal pivot for MCTS application may be an important issue. Therefore, we choose the diff_cut method with 0.75 as our final choice in the proposed work.

### 5.4. Modified CVRP Problems

In this section, we report the performance results for a modified CVRP to demonstrate the flexibility of our proposed method. For this purpose, we consider two cases, vehicle refilling amount and multiple depots, in which neither the classical formulation nor its heuristic solvers apply in their original form. To solve these cases with a classical formulation, we have to reconstruct the formulation from scratch. Solving these cases with heuristic solvers also requires reformulation. Meanwhile, our proposed method only needs one pre-training of the AM per modified CVRP problem, rather than reformulating the entire problem. Therefore, only the results of our proposed methods are provided here since classical formulations and heuristic solvers require reformulating the entire problem. Obviously, the results will be different from those in previous sections. We select two difference cut hyper-parameters, 0.25 and 0.75, use SwiGLU as the activation function, and set the total number of nodes *n* to 100.

In the first case, when visiting the depot node, we vary the refilling amount from 1 to 0.8 and 1.2, while the standard refilling amount for all other experiments is 1. We pretrain the AM for each refilling amount. As the refill amount decreases, the optimal solution should have a higher score because the vehicle must visit the depot node more frequently. We expect our model’s solution to exhibit this tendency. As expected, Table 13 shows that our model is able to find better solutions when the refill amount is larger, indicating that our proposed method is robust and capable of handling changes in the refill amount. Moreover, as the refill amount increases, the application of MCTS improves performance.

In the second case, we examine the performance of the modified CVRP problem with three depot nodes. We pretrain the AM for each number of depot nodes. As the number of depot nodes increases, the score of the optimal solution should decrease because the vehicle has more flexibility to choose a depot node when refilling is required. As expected, Table 14 shows that our model is able to find better solutions for more depot nodes, demonstrating that our model is robust to changes in the number of depot nodes. However, it is difficult to conclude that the application of MCTS results in better results compared to the use of AM in multi-depot CVRP problems since only a few MCTS-application cases show significant improvement.

In summary, our proposed method is robust to changes in CVRP formulation and sufficiently flexible to apply to modified problem formulations. However, the application of MCTS improves the solution for the vehicle-refill change, while its results remain unclear for multiple depot changes.

## 6. Conclusions and Future Works

We applied MCTS selectively in routing problems to determine whether it generated better solutions. Although the performance was still inferior to heuristic solvers, applying MCTS did generate better solutions than the case without MCTS. We also confirmed that using SwiGLU activation rather than typical ReLU can produce better solutions. The results of the baseline experiment remain controversial, but using mean-over-batches baselines generally helps generate better solutions. We believe that applying MCTS to different VRP problems with more complex situations may reveal the efficacy of the value-network-based baseline.

Future works can be developed from this paper. Firstly, the runtime of applying MCTS can be reduced if it is implemented in C++, which is much faster than Python. Also, if it is implemented in C++ (6.0), a parallelized version of MCTS [44] could help boost simulation time. Second, MCTS can be extended to other NP-hard problems in the field, e.g., bin-packing and knap-sack. Third, it is worth checking whether MCTS helps generalization across different problem sizes. Besides the changes in the refill amounts and depot numbers, it is also worth reflecting on other meaningful changes that real-world VRP may face, such as changes in traffic conditions or vehicle availability.

## Figures and Tables

**Table 1:** Results of TSP problems.

Problem Size (*n*)				20		50		100	
Method	Activation	Baseline	ns	Dist	Time	Dist	Time	Dist	Time
LKH3	N.A.			3.8402 ± 0.05	0.04 ± 0.01	5.6705 ± 0.05	0.411 ± 0.07	7.7352 ± 0.05	1.167 ± 0.24
OR Tools				3.8402 ± 0.30	1.001 ± 0.00	5.6807 ± 0.24	1.00 ± 0.00	7.9003 ± 0.28	7.001 ± 0.00
Nearest Insertions				4.3742 ± 0.08	0.001 ± 0.00	6.7550 ± 0.07	0.00 ± 0.00	9.4517 ± 0.08	0.003 ± 0.00
Concorde				3.8402 ± 0.05	0.124 ± 0.03	5.6705 ± 0.05	1.292 ± 0.23	7.7352 ± 0.05	5.143 ± 0.69
DRL	ReLU	mean	0	3.8492 ± 0.09	0.037 ± 0.00	5.7361 ± 0.06	0.079 ± 0.00	7.9852 ± 0.08	0.153 ± 0.00
			100	**3.8486 ± 0.09**	0.043 ± 0.00	5.7375 ± 0.06	0.205 ± 0.01	7.9854 ± 0.08	0.627 ± 0.06
			500	3.8491 ± 0.09	0.059 ± 0.00	5.7345 ± 0.06	0.592 ± 0.23	7.9826 ± 0.08	2.588 ± 1.82
			1000	3.8491 ± 0.09	0.077 ± 0.01	**5.7339 ± 0.06**	1.051 ± 0.85	**7.9807 ± 0.08**	5.509 ± 9.14
		val	0	3.8489 ± 0.08	0.038 ± 0.00	5.7386 ± 0.06	0.078 ± 0.00	8.0481 ± 0.08	0.154 ± 0.00
			100	**3.8483 ± 0.08**	0.038 ± 0.00	5.7310 ± 0.07	0.194 ± 0.01	**8.0465 ± 0.08**	0.648 ± 0.08
			500	**3.8483 ± 0.08**	0.044 ± 0.00	**5.7291 ± 0.07**	0.533 ± 0.21	8.0687 ± 0.09	2.897 ± 2.03
			1000	**3.8483 ± 0.08**	0.052 ± 0.01	5.7301 ± 0.06	0.959 ± 0.88	8.0564 ± 0.08	5.915 ± 9.45
	SwiGLU	mean	0	3.8464 ± 0.08	0.037 ± 0.00	5.7267 ± 0.07	0.080 ± 0.00	7.9562 ± 0.07	0.155 ± 0.00
			100	3.8462 ± 0.08	0.045 ± 0.00	**5.7248 ± 0.07**	0.173 ± 0.01	**7.9523 ± 0.07**	0.594 ± 0.09
			500	**3.8461 ± 0.08**	0.065 ± 0.01	5.7260 ± 0.07	0.458 ± 0.17	7.9530 ± 0.07	2.362 ± 2.72
			1000	**3.8461 ± 0.08**	0.089 ± 0.02	5.7257 ± 0.07	0.799 ± 0.62	7.9539 ± 0.07	4.941 ± 13.23
		val	0	3.8482 ± 0.09	0.038 ± 0.00	5.7405 ± 0.07	0.081 ± 0.00	7.9551 ± 0.07	0.156 ± 0.00
			100	**3.8474 ± 0.09**	0.047 ± 0.00	5.7377 ± 0.07	0.192 ± 0.01	7.9536 ± 0.07	0.646 ± 0.07
			500	3.8478 ± 0.09	0.069 ± 0.01	5.7377 ± 0.07	0.550 ± 0.19	7.9630 ± 0.07	2.781 ± 1.93
			1000	3.8478 ± 0.09	0.093 ± 0.02	**5.7373 ± 0.07**	0.961 ± 0.70	**7.9522 ± 0.07**	5.822 ± 9.99

**Table 2:** CVRP problem result.

Problem Size (*n*)				20		50		100	
Method	Activation	Baseline	ns	Dist	Time	Dist	Time	Dist	Time
LKH3	N.A.			6.1528 ± 0.16	3.948 ± 0.46	10.2951 ± 0.24	14.617 ± 1.15	15.4804 ± 0.33	26.03 ± 1.87
OR Tools				6.2049 ± 0.85	1.002 ± 0.00	10.5973 ± 1.25	5.000 ± 0.00	16.273 ± 1.76	18.000 ± 0
DRL	ReLU	mean	0	6.4097 ± 0.75	0.045 ± 0.00	**10.8050 ± 1.65**	0.095 ± 0.00	16.4418 ± 2.99	0.178 ± 0.00
			100	**6.4010 ± 0.76**	0.151 ± 0.01	10.8225 ± 1.59	0.473 ± 0.03	16.4627 ± 3.00	1.326 ± 0.12
			500	6.4065 ± 0.75	0.398 ± 0.13	10.8192 ± 1.60	1.737 ± 0.81	16.4596 ± 2.99	6.652 ± 3.91
			1000	6.4073 ± 0.76	0.685 ± 0.45	10.8087 ± 1.63	3.258 ± 2.95	**16.4387 ± 2.96**	13.633 ± 17.61
		value	0	6.4553 ± 0.80	0.046 ± 0.00	**10.8634 ± 1.65**	0.095 ± 0.00	**16.4463 ± 3.00**	0.177 ± 0.00
			100	6.4452 ± 0.83	0.176 ± 0.01	10.8718 ± 1.61	0.466 ± 0.03	16.4750 ± 3.08	1.409 ± 0.13
			500	**6.4449 ± 0.84**	0.489 ± 0.15	10.8651 ± 1.65	1.773 ± 0.78	16.4494 ± 3.02	7.079 ± 3.34
			1000	6.4479 ± 0.80	0.831 ± 0.47	10.8774 ± 1.67	3.475 ± 3.65	16.4468 ± 2.98	14.504 ± 14.40
	SwiGLU	mean	0	6.4231 ± 0.73	0.046 ± 0.00	10.7940 ± 1.70	0.097 ± 0.00	**16.4043 ± 3.09**	0.180 ± 0.00
			100	**6.4228 ± 0.75**	0.141 ± 0.01	**10.7747 ± 1.71**	0.464 ± 0.05	16.4190 ± 3.10	1.243 ± 0.14
			500	6.4230 ± 0.73	0.346 ± 0.08	10.7878 ± 1.72	1.742 ± 1.02	16.4169 ± 3.10	6.018 ± 4.62
			1000	6.4292 ± 0.74	0.562 ± 0.23	10.7847 ± 1.67	3.260 ± 3.71	16.4117 ± 3.11	12.221 ± 20.71
		val	0	6.4201 ± 0.72	0.046 ± 0.00	10.8402 ± 1.48	0.096 ± 0.00	16.3575 ± 3.04	0.178 ± 0.00
			100	**6.4020 ± 0.74**	0.154 ± 0.01	10.8420 ± 1.53	0.517 ± 0.04	16.3950 ± 2.97	1.188 ± 0.11
			500	6.4055 ± 0.73	0.422 ± 0.10	**10.8336 ± 1.49**	1.959 ± 1.02	**16.3475 ± 3.05**	5.671 ± 3.51
			1000	6.4101 ± 0.72	0.686 ± 0.28	10.8456 ± 1.53	3.683 ± 3.64	16.3492 ± 3.02	11.610 ± 14.31

**Table 3:** A *t*-test report with the same conditions.

Problem Type	Problem Size	AM	MCTS	*p*-Value	<0.05
tsp	20	SwiGLU-val	SwiGLU-val-100	0.1	FALSE
	50	ReLU-val	ReLU-val-500	0.0144	TRUE
	100	ReLU-mean	ReLU-mean-1000	0.0262	TRUE
cvrp	20	SwiGLU-val	SwiGLU-val-1000	0.0066	TRUE
	50	SwiGLU-mean	SwiGLUe-mean-100	0.0994	FALSE
	100	SwiGLU-val	SwiGLU-val-100	0.0339	TRUE

**Table 4:** A *t*-test report regardless of the conditions.

Problem Type	Problem Size	AM	MCTS	*p*-Value	<0.05
tsp	20	SwiGLU-val	SwiGLU-val-100	0.1	FALSE
	50	ReLU-val	ReLU-val-500	0.0144	TRUE
	100	ReLU-val	SwiGLU-mean-100	0.0001	TRUE
cvrp	20	SwiGLU-val	SwiGLU-val-1000	0.0066	TRUE
	50	ReLU-val	SwiGLUe-mean-100	0.0064	TRUE
	100	SwiGLU-val	ReLU-mean-100	0.0056	TRUE

**Table 5:** Performance results according to activation functions.

Problem	Size	20		50		100	
Type	Activation	Score	Runtime	Score	Runtime	Score	Runtime
**TSP**	**ReLU**	3.8487 ± 0.09	0.049 ± 0.00	5.7338 ± 0.06	0.462 ± 0.27	8.0192 ± 0.08	2.312 ± 2.82
	**SwiGLU**	**3.8470 ± 0.08**	0.060 ± 0.01	**5.7321 ± 0.07**	0.412 ± 0.21	**7.9549 ± 0.07**	2.182 ± 3.50
**CVRP**	**ReLU**	6.4272 ± 0.79	0.353 ± 0.15	10.8416 ± 1.63	1.421 ± 1.03	16.4526 ± 3.00	5.620 ± 4.94
	**SwiGLU**	**6.4170 ± 0.73**	0.300 ± 0.09	**10.8128 ± 1.60**	1.477 ± 1.18	**16.3876 ± 3.06**	4.789 ± 5.43

**Table 6:** Aggregated results of baselines.

Problem	Size	20		50		100	
Type	Baseline	Score	Runtime	score	Runtime	Score	Runtime
**TSP**	**mean**	**3.8476 ± 0.09**	0.057 ± 0.01	**5.7306 ± 0.06**	0.429 ± 0.24	**7.9687 ± 0.08**	2.116 ± 3.38
	**value**	3.8481 ± 0.08	0.052 ± 0.00	5.7352 ± 0.07	0.444 ± 0.25	8.0054 ± 0.08	2.378 ± 2.94
**CVRP**	**mean**	**6.4153 ± 0.75**	0.297 ± 0.11	**10.7996 ± 1.66**	1.391 ± 1.07	16.4319 ± 3.04	5.181 ± 5.89
	**value**	6.4289 ± 0.77	0.356 ± 0.13	10.8549 ± 1.57	1.508 ± 1.15	**16.4083 ± 3.02**	5.227 ± 4.48

**Table 7:** Non-selective MCTS applied on TSP.

Problem Size				20		50		100	
Method	Activation	Baseline	ns	Dist	Time	Dist	Time	Dist	Time
LKH3	N.A.			3.8402 ± 0.05	0.04± 0.01	5.6705 ± 0.05	0.411 ± 0.07	7.7352 ± 0.05	1.167 ± 0.24
OR Tools				3.8402 ± 0.30	5.001 ± 0.00	5.6807 ± 0.24	5.00 ± 0.00	7.9154 ± 0.28	5.001 ± 0.00
Neareset Insertions				4.3742 ± 0.08	0.001 ± 0.00	6.7550 ± 0.07	0.00 ± 0.00	9.4517 ± 0.08	0.003 ± 0.00
Concorde				3.8402 ± 0.05	0.124 ± 0.03	5.6705 ± 0.05	1.292 ± 0.23	7.7352 ± 0.05	5.143 ± 0.69
DRL	ReLU	mean	0	3.8492 ± 0.09	0.107 ± 0.00	5.7361 ± 0.06	0.231 ± 0.00	7.9852 ± 0.08	0.450 ± 0.00
			100	**3.8491 ± 0.09**	0.864 ± 0.03	**5.7356 ± 0.06**	4.614 ± 0.55	**7.9817 ± 0.08**	10.342 ± 2.31
			500	**3.8491 ± 0.09**	3.079 ± 0.25	5.7365 ± 0.06	17.099 ± 2.76	7.9855 ± 0.08	55.007 ± 24.24
			1000	**3.8491 ± 0.09**	5.727 ± 0.34	5.7360 ± 0.06	31.821 ± 5.26	7.9848 ± 0.08	110.943 ± 76.95
		value	0	3.8489 ± 0.08	0.111 ± 0.00	5.7386 ± 0.06	0.230 ± 0.00	8.0481 ± 0.08	0.451 ± 0.00
			100	3.8489 ± 0.08	0.879 ± 0.07	**5.7294 ± 0.07**	4.450 ± 0.60	8.0459 ± 0.08	9.434 ± 3.04
			500	**3.8483 ± 0.08**	2.962 ± 0.08	5.7362 ± 0.06	16.844 ± 3.02	**8.0453 ± 0.08**	56.209 ± 35.65
			1000	**3.8483 ± 0.08**	5.715 ± 0.28	5.7314 ± 0.07	31.341 ± 4.81	8.0456 ± 0.08	112.687 ± 105.85
	SwiGLU	mean	0	3.8464 ± 0.08	0.110 ± 0.00	5.7267 ± 0.07	0.237 ± 0.00	7.9562 ± 0.07	0.454 ± 0.00
			100	3.8463 ± 0.08	0.940 ± 0.13	**5.7263 ± 0.07**	4.292 ± 0.38	**7.9528 ± 0.07**	10.239 ± 3.44
			500	**3.8459 ± 0.08**	2.979 ± 0.12	5.7266 ± 0.07	16.516 ± 1.83	7.9542 ± 0.07	56.392 ± 29.80
			1000	3.8461 ± 0.08	5.716 ± 0.32	5.7265 ± 0.07	30.746 ± 2.69	7.9542 ± 0.07	112.849 ± 84.50
		value	0	3.8482 ± 0.09	0.113 ± 0.00	5.7405 ± 0.07	0.234 ± 0.00	7.9551 ± 0.07	0.456 ± 0.00
			100	**3.8481 ± 0.09**	0.860 ± 0.04	5.7403 ± 0.07	4.429 ± 0.45	**7.9509 ± 0.07**	10.046 ± 3.79
			500	3.8481 ± 0.09	3.040 ± 0.16	**5.7391 ± 0.07**	16.917 ± 2.96	7.9516 ± 0.07	55.425 ± 39.04
			1000	3.8481 ± 0.09	5.705 ± 0.27	**5.7391 ± 0.07**	31.496 ± 4.65	7.9516 ± 0.07	111.637 ± 113.14

**Table 8:** Non-selective MCTS applied on CVRP.

Problem Size				20		50		100	
Method	Activation	Baseline	ns	Dist	Time	Dist	Time	Dist	Time
LKH3	N.A.			6.1528 ± 0.16	3.948 ± 0.46	10.2951 ± 0.24	14.617 ± 1.15	15.4804 ± 0.33	26.03 ± 1.87
OR Tools				6.2049 ± 0.85	1.002 ± 0.00	10.5973 ± 1.25	5.000 ± 0.00	16.273 ± 1.76	18.000 ± 0
DRL	ReLU	mean	0	6.4097 ± 0.75	0.131 ± 0.00	**10.8050 ± 1.65**	0.279 ± 0.00	16.4418 ± 2.99	0.523 ± 0.00
			100	**6.4048 ± 0.77**	1.067 ± 0.16	10.8133 ± 1.60	3.444 ± 0.57	16.4840 ± 3.06	6.453 ± 2.58
			500	6.4096 ± 0.77	3.258 ± 1.10	10.8100 ± 1.59	14.640 ± 6.72	16.4517 ± 2.99	35.803 ± 47.22
			1000	6.4230 ± 0.79	5.631 ± 2.54	10.8113 ± 1.64	27.046 ± 20.52	**16.4454 ± 3.01**	73.464 ± 156.85
		value	0	6.4553 ± 0.80	0.132 ± 0.00	**10.8634 ± 1.65**	0.276 ± 0.00	**16.4463 ± 3.00**	0.525 ± 0.00
			100	6.4425 ± 0.83	1.185 ± 0.19	10.8717 ± 1.62	3.426 ± 0.50	16.4812 ± 3.08	6.455 ± 2.61
			500	**6.4412 ± 0.82**	3.515 ± 1.18	10.8708 ± 1.66	14.678 ± 6.82	16.4507 ± 3.02	35.825 ± 42.25
			1000	6.4475 ± 0.81	5.890 ± 2.85	10.8715 ± 1.67	27.310 ± 20.23	16.4530 ± 2.98	73.602 ± 166.00
	SwiGLU	mean	0	6.4231 ± 0.73	0.133 ± 0.00	10.7940 ± 1.70	0.278 ± 0.00	**16.4043 ± 3.09**	0.527 ± 0.00
			100	**6.4226 ± 0.75**	1.047 ± 0.15	**10.7908 ± 1.72**	3.371 ± 0.53	16.4193 ± 3.11	6.441 ± 3.31
			500	6.4248 ± 0.73	3.051 ± 0.63	10.7959 ± 1.71	14.491 ± 7.00	16.4164 ± 3.11	35.644 ± 49.24
			1000	6.4333 ± 0.75	5.338 ± 1.48	10.7933 ± 1.69	26.904 ± 22.83	16.4148 ± 3.13	73.034 ± 205.68
		value	0	6.4201 ± 0.72	0.132 ± 0.00	10.8402 ± 1.48	0.277 ± 0.00	16.3575 ± 3.04	0.525 ± 0.00
			100	**6.4047 ± 0.75**	1.129 ± 0.21	10.8389 ± 1.52	3.560 ± 0.66	16.3928 ± 3.01	6.475 ± 2.65
			500	6.4105 ± 0.74	3.314 ± 0.84	**10.8328 ± 1.49**	15.411 ± 7.90	**16.3479 ± 3.04**	35.775 ± 52.90
			1000	6.4138 ± 0.73	5.554 ± 1.49	10.8422 ± 1.51	28.610 ± 27.21	16.3497 ± 3.03	72.945 ± 187.09

**Table 9:** TSP 100 difference pivot result.

Activation	Baseline	ns	diff_cut = 0.10		diff_cut = 0.25		diff_cut = 0.50		diff_cut = 0.75	
			Score	Runtime	Score	Runtime	Score	Runtime	Score	Runtime
**SwiGLU**	**mean**	**0**	7.9562 ± 0.07	0.121 ± 0.00	7.9562 ± 0.07	0.121 ± 0.00	7.9562 ± 0.07	0.121 ± 0.00	7.9562 ± 0.07	0.121 ± 0.00
		**100**	**7.9541 ± 0.07**	0.124 ± 0.00	**7.9541 ± 0.07**	0.124 ± 0.00	7.9541 ± 0.07	0.135 ± 0.00	**7.9523 ± 0.07**	0.621 ± 0.13
		**500**	**7.9541 ± 0.07**	0.124 ± 0.00	**7.9541 ± 0.07**	0.124 ± 0.00	7.9554 ± 0.07	0.198 ± 0.05	7.9530 ± 0.07	2.586 ± 3.31
		**1000**	**7.9541 ± 0.07**	0.126 ± 0.00	**7.9541 ± 0.07**	0.126 ± 0.00	**7.9540 ± 0.07**	0.283 ± 0.21	7.9539 ± 0.07	5.553 ± 18.05
	**value**	**0**	7.9551 ± 0.07	0.156 ± 0.00	7.9551 ± 0.07	0.156 ± 0.00	7.9551 ± 0.07	0.156 ± 0.00	7.9551 ± 0.07	0.156 ± 0.00
		**100**	**7.9544 ± 0.07**	0.123 ± 0.00	**7.9544 ± 0.07**	0.123 ± 0.00	7.9547 ± 0.07	0.143 ± 0.00	7.9536 ± 0.07	0.679 ± 0.12
		**500**	**7.9544 ± 0.07**	0.123 ± 0.00	**7.9544 ± 0.07**	0.123 ± 0.00	**7.9527 ± 0.07**	0.244 ± 0.07	7.9630 ± 0.07	3.114 ± 3.52
		**1000**	**7.9544 ± 0.07**	0.124 ± 0.00	**7.9544 ± 0.07**	0.124 ± 0.00	7.9528 ± 0.07	0.400 ± 0.37	**7.9522 ± 0.07**	6.532 ± 17.42

**Table 10:** CVRP 100 difference pivot result.

Activation	Baseline	ns	diff_cut = 0.10		diff_cut = 0.25		diff_cut = 0.5		diff_cut = 0.75	
			Score	Runtime	Score	Runtime	Score	Runtime	Score	Runtime
**SwiGLU**	**mean**	**0**	**16.4043 ± 3.09**	0.142 ± 0.00	**16.4043 ± 3.09**	0.142 ± 0.00	**16.4043 ± 3.09**	0.142 ± 0.00	**16.4043 ± 3.09**	0.142 ± 0.00
		**100**	**16.4043 ± 3.09**	0.351 ± 0.01	**16.4043 ± 3.09**	0.293 ± 0.01	16.4132 ± 3.09	0.213 ± 0.01	16.4190 ± 3.10	1.253 ± 0.15
		**500**	**16.4043 ± 3.09**	0.329 ± 0.00	**16.4043 ± 3.09**	0.310 ± 0.03	16.4095 ± 3.11	0.528 ± 0.25	16.4169 ± 3.10	6.293 ± 5.36
		**1000**	**16.4043 ± 3.09**	0.335 ± 0.01	**16.4043 ± 3.09**	0.346 ± 0.16	16.4115 ± 3.12	0.931 ± 1.06	16.4117 ± 3.11	12.700 ± 23.47
	**value**	**0**	**16.3575 ± 3.04**	0.142 ± 0.00	**16.3575 ± 3.04**	0.142 ± 0.00	16.3575 ± 3.04	0.142 ± 0.00	16.3575 ± 3.04	0.142 ± 0.00
		**100**	16.3595 ± 3.05	0.341 ± 0.01	16.3597 ± 3.05	0.301 ± 0.01	**16.3536 ± 2.98**	0.217 ± 0.01	16.3950 ± 2.97	1.201 ± 0.12
		**500**	16.3595 ± 3.05	0.327 ± 0.00	16.3597 ± 3.05	0.318 ± 0.04	16.3549 ± 3.04	0.541 ± 0.27	**16.3475 ± 3.05**	5.947 ± 4.08
		**1000**	16.3595 ± 3.05	0.343 ± 0.01	16.3597 ± 3.05	0.333 ± 0.10	16.3551 ± 3.04	0.952 ± 1.14	16.3492 ± 3.02	12.072 ± 16.35

**Table 11:** Results for TSP 100 with entropy pivots.

Activation	Baseline	ns	ent_cut = 0.25		ent_cut = 0.5		ent_cut = 0.75		ent_cut = 1	
			Score	Runtime	Score	Runtime	Score	Runtime	Score	Runtime
**SwiGLU**	**mean**	**0**	7.9562 ± 0.07	0.123 ± 0.00	7.9562 ± 0.07	0.123 ± 0.00	7.9562 ± 0.07	0.123 ± 0.00	7.9562 ± 0.07	0.123 ± 0.00
		**100**	**7.9532 ± 0.07**	1.180 ± 0.21	**7.9532 ± 0.07**	0.499 ± 0.04	**7.9541 ± 0.07**	0.358 ± 0.02	**7.9541 ± 0.07**	0.314 ± 0.01
		**500**	7.9544 ± 0.07	5.739 ± 7.98	7.9542 ± 0.07	1.681 ± 1.16	7.9553 ± 0.07	0.728 ± 0.67	7.9554 ± 0.07	0.496 ± 0.25
		**1000**	7.9543 ± 0.07	12.532 ± 41.43	7.9543 ± 0.07	3.405 ± 5.95	7.9543 ± 0.07	1.277 ± 3.42	7.9547 ± 0.07	0.729 ± 1.12
	**value**	**0**	7.9551 ± 0.07	0.124 ± 0.00	7.9551 ± 0.07	0.124 ± 0.00	7.9551 ± 0.07	0.124 ± 0.00	7.9551 ± 0.07	0.124 ± 0.00
		**100**	7.9537 ± 0.07	1.361 ± 0.25	7.9541 ± 0.07	0.556 ± 0.04	7.9554 ± 0.07	0.394 ± 0.03	7.9547 ± 0.07	0.317 ± 0.01
		**500**	7.9578 ± 0.07	6.963 ± 8.41	7.9584 ± 0.07	2.081 ± 1.45	**7.9506 ± 0.07**	0.925 ± 0.94	7.9527 ± 0.07	0.576 ± 0.41
		**1000**	**7.9523 ± 0.07**	15.041 ± 43.92	**7.9529 ± 0.07**	4.243 ± 6.65	7.9514 ± 0.07	1.711 ± 4.56	**7.9525 ± 0.07**	0.867 ± 1.67

**Table 12:** Results for CVRP 100 with entropy pivots.

Activation	Baseline	ns	ent_cut = 0.25		ent_cut = 0.5		ent_cut = 0.75		ent_cut = 1	
			Score	Runtime	Score	Runtime	Score	Runtime	Score	Runtime
**SwiGLU**	**mean**	**0**	**16.4043 ± 3.09**	0.180 ± 0.00	**16.4043 ± 3.09**	0.180 ± 0.00	**16.4043 ± 3.09**	0.180 ± 0.00	**16.4043 ± 3.09**	0.180 ± 0.00
		**100**	16.4242 ± 3.09	2.854 ± 0.63	16.4164 ± 3.09	0.910 ± 0.10	16.4145 ± 3.07	0.441 ± 0.08	16.4143 ± 3.08	0.218 ± 0.01
		**500**	16.4106 ± 3.11	16.915 ± 20.97	16.4118 ± 3.10	4.370 ± 2.99	16.4146 ± 3.11	1.578 ± 2.71	16.4130 ± 3.11	0.547 ± 0.22
		**1000**	16.4100 ± 3.11	34.895 ± 87.57	16.4100 ± 3.11	8.889 ± 13.80	16.4120 ± 3.12	3.125 ± 11.77	16.4088 ± 3.12	0.974 ± 0.93
	**value**	**0**	16.3575 ± 3.04	0.178 ± 0.00	16.3575 ± 3.04	0.178 ± 0.00	16.3575 ± 3.04	0.178 ± 0.00	16.3575 ± 3.04	0.178 ± 0.00
		**100**	16.3839 ± 3.05	2.730 ± 0.61	16.3802 ± 3.02	0.899 ± 0.11	16.3680 ± 3.02	0.459 ± 0.08	16.3666 ± 3.03	0.232 ± 0.01
		**500**	16.3561 ± 3.06	15.386 ± 15.60	**16.3530 ± 3.05**	4.338 ± 2.80	16.3575 ± 3.06	1.658 ± 1.89	16.3583 ± 3.05	0.647 ± 0.29
		**1000**	**16.3558 ± 3.04**	31.816 ± 66.79	16.3531 ± 3.03	8.700 ± 11.07	**16.3573 ± 3.04**	3.256 ± 8.14	**16.3572 ± 3.04**	1.168 ± 1.18

**Table 13:** Performance for vehicle refills.

Refill Amount				0.8		1		1.2	
Method	ent_cut	Baseline	ns	Score	Runtime	Score	Runtime	Score	Runtime
SwiGLU	0.25	mean	0	**19.5630 ± 5.29**	0.140 ± 0.00	**16.4043 ± 3.09**	0.142 ± 0.00	14.8674 ± 2.94	0.133 ± 0.00
			100	19.5774 ± 5.36	0.166 ± 0.00	**16.4043 ± 3.09**	0.293 ± 0.00	14.8581 ± 2.78	0.146 ± 0.00
			500	19.5735 ± 5.36	0.328 ± 0.18	**16.4043 ± 3.09**	0.310 ± 0.03	14.8632 ± 2.79	0.196 ± 0.08
			1000	19.5733 ± 5.36	0.513 ± 0.84	**16.4043 ± 3.09**	0.346 ± 0.16	**14.8567 ± 2.76**	0.253 ± 0.26
		value	0	**19.9787 ± 5.84**	0.138 ± 0.00	**16.3575 ± 3.03**	0.142 ± 0.00	14.7268 ± 1.96	0.133 ± 0.00
			100	20.0338 ± 5.78	0.232 ± 0.01	16.3597 ± 3.05	0.301 ± 0.01	14.7260 ± 1.96	0.136 ± 0.00
			500	20.0108 ± 5.83	0.715 ± 0.45	16.3597 ± 3.05	0.318 ± 0.04	**14.7253 ± 1.96**	0.141 ± 0.00
			1000	20.0072 ± 5.85	1.306 ± 1.92	16.3597 ± 3.05	0.333 ± 0.10	**14.7253 ± 1.96**	0.146 ± 0.01
	0.75	mean	0	**19.5630 ± 5.29**	0.140 ± 0.00	**16.4043 ± 3.09**	0.142 ± 0.00	14.8674 ± 2.94	0.133 ± 0.00
			100	19.6385 ± 5.14	1.369 ± 0.13	16.4190 ± 3.10	1.253 ± 0.15	14.8840 ± 2.80	1.073 ± 0.17
			500	19.6096 ± 5.43	7.649 ± 4.39	16.4169 ± 3.10	6.293 ± 5.36	**14.8629 ± 2.78**	6.567 ± 5.25
			1000	19.6208 ± 5.39	16.177 ± 19.05	16.4117 ± 3.11	12.700 ± 23.47	14.8810 ± 2.81	14.262 ± 26.05
		value	0	**19.9787 ± 5.84**	0.138 ± 0.00	16.3575 ± 3.04	0.142 ± 0.00	14.7268 ± 1.96	0.133 ± 0.00
			100	20.0404 ± 6.18	1.768 ± 0.18	16.3950 ± 2.97	1.201 ± 0.12	14.7533 ± 2.00	1.117 ± 0.10
			500	20.0736 ± 6.29	10.515 ± 6.04	**16.3475 ± 4.05**	5.947 ± 4.08	**14.7204 ± 1.94**	6.996 ± 3.75
			1000	20.0147 ± 5.67	22.416 ± 26.62	16.3492 ± 3.02	12.072 ± 16.35	14.7116 ± 1.97	15.164 ± 16.69

**Table 14:** Performance for multiple depot nodes.

Number of Depot Nodes				1		3	
Method	diff_cut	Baseline	ns	Score	Runtime	Score	Runtime
SwiGLU	0.25	mean	0	**16.4043 ± 3.09**	0.142 ± 0.00	**15.5005 ± 2.54**	0.136 ± 0.00
			100	**16.4043 ± 3.09**	0.293 ± 0.00	15.5076 ± 2.56	0.139 ± 0.00
			500	**16.4043 ± 3.09**	0.310 ± 0.03	15.5076 ± 2.56	0.164 ± 0.02
			1000	**16.4043 ± 3.09**	0.346 ± 0.16	15.5077 ± 2.56	0.197 ± 0.08
		value	0	**16.3575 ± 3.03**	0.142 ± 0.00	15.5699 ± 2.61	0.135 ± 0.00
			100	16.3597 ± 3.05	0.301 ± 0.01	15.5754 ± 2.62	0.139 ± 0.00
			500	16.3597 ± 3.05	0.318 ± 0.04	**15.5696 ± 2.61**	0.163 ± 0.01
			1000	16.3597 ± 3.05	0.333 ± 0.10	**15.5696 ± 2.61**	0.195 ± 0.06
	0.75	mean	0	**16.4043 ± 3.09**	0.142 ± 0.00	**15.5005 ± 2.54**	0.136 ± 0.00
			100	16.4190 ± 3.10	1.253 ± 0.15	15.5638 ± 2.81	1.066 ± 0.11
			500	16.4169 ± 3.10	6.293 ± 5.36	15.5298 ± 2.63	6.209 ± 4.21
			1000	16.4117 ± 3.11	12.700 ± 23.47	15.5026 ± 2.57	13.324 ± 18.63
		value	0	16.3575 ± 3.04	0.142 ± 0.00	15.5699 ± 2.61	0.135 ± 0.00
			100	16.3950 ± 2.97	1.201 ± 0.12	**15.5163 ± 2.56**	1.072 ± 0.08
			500	**16.3475 ± 4.05**	5.947 ± 4.08	15.5701 ± 2.73	6.516 ± 3.38
			1000	16.3492 ± 3.02	12.072 ± 16.35	15.5553 ± 2.67	14.033 ± 15.54

## Data Availability

The data will be available upon request.
